# Electrocardiogram of Clinically Healthy Mithun (*Bos frontalis*): Variation among Strains

**DOI:** 10.4061/2010/790310

**Published:** 2010-09-22

**Authors:** Sagar Sanyal, Pradip Kumar Das, Probal Ranjan Ghosh, Kinsuk Das, Kezha V. Vupru, Chandan Rajkhowa, Mohan Mondal

**Affiliations:** ^1^Department of Veterinary Physiology, Faculty of Veterinary and Animal Sciences, West Bengal University of Animal and Fishery Sciences, 37, Kshudiram Bose Sarani, Kolkata 700 037, India; ^2^National Research Centre on Mithun, Indian Council of Agricultural Research Jharnapani, Medziphema, Nagaland 797106, India

## Abstract

A study was conducted to establish the normal electrocardiogram in four different genetic strains of mithun (*Bos frontalis*). Electrocardiography, cardiac electrical axis, heart rate, rectal temperature and respiration rate were recorded in a total of 32 adult male mithun of four strains (*n* = 8 each). It was found that the respiration and heart rates were higher (*P* < .05) in Manipur than other three strains. Amplitude (*P* < .05) and duration of P wave and QRS complex differed (*P* < .01) among the strains. Mizoram strain had the highest amplitude and duration of P wave and QRS complex. On the other hand, higher (*P* < .05) amplitude and duration of T wave were recorded in Arunachalee and Mizoram strains. The mean electrical axis of QRS complex that were recorded for Arunachalee and Manipur strains were similar to that reported for other bovine species; whereas the electrical axis of QRS for Nagamese and Mizoram strains were more close to feline and caprine species, respectively. In conclusion, electrocardiogram of mithun revealed that the amplitude and duration of P wave, QRS complex and T wave were different among four different genetic strains of mithun and the electrical axis of QRS complex for Nagamese and Mizoram mithuns are dissimilar to bovine species.

## 1. Introduction

Mithun (*Bos frontalis*), a semi-domesticated rare ruminant of South-east Asia, is believed to have originated more than 8000 years ago and is considered to be a descendent from wild Indian gaur [1]. This prized hill animal is found mainly in four different States of the North-Eastern Hill region (NEHR) of India, namely, Arunachal Pradesh, Nagaland, Manipur, and Mizoram having different geographical locations and also in some locations of Bhutan, Myanmar, Bangladesh, and China. Mithuns from four different States of NEHR of India are reported to be genetically different from each other, and each strain of mithun is named as per the state where from they originated [2]. This massive hill animal of NEHR plays an important role in the socioeconomic and cultural life of the local population, beside it acts as a potential source of meat [1]. However, due to remoteness of their habitats and other ecological and socioeconomic factors, mithuns remain one of the least studied ungulates.

In NEHR of India, mithun meat is considered to be more tender and superior over the meat of any other species. At present, mithun farmers rear this animal at an altitude of 1000 to 3000 meters above mean sea level under free grazing condition in its natural habitat. Due to gradual denudation of forests (natural habitat of mithun) and tremendous socioeconomic and cultural importance of mithun in the life of the local tribal population, very recently initiatives are being taken to popularise economic mithun farming under semi-intensive condition with an effort to make use of mithun for benefit of human population. 

The mithun normally lives in the areas where low oxygen tension prevails and thrives on very little conventional fodder. This animal is bestowed with an excellent capacity to adjust in its natural climate and to graze on stiff hill slopes with ease. In congruence with this unique characteristic, some physiological adaptations especially on the cardiovascular system are envisaged. The exploration of the exact cardiovascular physiology has profound implications on mapping of fatigue score and draught power as well as various fields of health and production management system. 

Electrocardiography is an inexpensive, noninvasive technique that entails useful information in classification of arrhythmias, diagnosing conduction abnormalities and also acts as a valuable aid in prognostic and therapeutic considerations [3–5]. The potential use of electrocardiogram (ECG) is well recognized in cattle [6, 7] and horse [8]. Electrical axis (EA) determined on QRS is a vector originating at the centre of Einthoven's triangle representing the direction of the ventricular activation process as projected in the plane of limb leads. EA provides important clues for derivation of ECG-pathological correlation and the degree of deviation refers to either ventricular activities or intermittent conduction disturbances [9]. Mean electrical axis traditionally applied to ventricular depolarization represents the average direction of electrical potential generated during cardiac cycle and is useful for suggesting chamber enlargement or intraventricular conduction defects [10, 11]. Like other ruminants, studies on ECG pattern vis-à-vis cardiac electrical axis of different strains of mithun would be helpful for diagnosis of ventricular conduction defects, ventricular hypertrophy, pulmonary embolism, obstructive lung diseases, and other associated haemodynamic abnormalities. As there is no information available on ECG in this species, the present study is hoped to serve as a scaffold in the field of electrophysiology of Mithun. Hence, the purpose of this study was to establish normal electrocardiogram in four different genetic strains of mithun using the standard bipolar limb leads (I, II, and III) and augmented unipolar limb leads (aVL, aVR, and aVF).

## 2. Materials And Methods

### 2.1. Study Area and Climate

The study was conducted at Medziphema Farm of the National Research Centre on Mithun, Indian Council of Agricultural Research, Nagaland located between 93.5°E and 25.5°N. The experiment was completed within 30 days. The mean (± SEM) atmospheric temperature, relative humidity, rain fall, and wind velocity during the experimental period was recorded to be 26.3 ± 3.1°C, 63.5 ± 0.9%, 3.2 ± 1.4 mm, and 24.2 ± 10.6 km/hour, respectively. 

### 2.2. Experimental Animals

A total of 32 healthy male mithuns from four different strains, namely, Nagamese, Arunachalee, Mizoram, and Manipur (*n* = 8 for each strain) with age and body weight ranged between 40 and 70 months, and 126 and 380 kg were selected for the present study after proper clinical examination and based on farm records. 

### 2.3. Experimental Parameters

The electrocardiogram (ECG) along with rectal temperature and respiration rate were measured in all animals. The rectal temperature was recorded twice a day at 0600 and 1700 h using electronic digital thermometer (OMRON, Japan). The respiration rate was also recorded. ECG was recorded by six-channel CARDIART—108 T/MK-VI (BPL, India) portable ECG machine as per standard procedure [12]. ECG tracings were recorded on standing posture. The machine was calibrated at 1mV=10 mm and paper speed of 25 mm/s. The right and left armed electrode were attached proximal to olecranon process on the caudal aspects of the appropriate four legs, whereas hind leg electrodes were attached over stifle joint on the anterior aspects of appropriate hind legs. The cardiac electrical axis of QRS of different strains of Mithun was determined from ECG using hexaxial reference system [10, 11]. Heart rate was calculated from ECG tracing by R-R method [13]. Amplitude and duration of P wave, QRS complex, and T wave were traced in all three standards bipolar and three unipolar leads. 

### 2.4. Statistical Analyses

Mean values and their standard errors of the mean (SEM) for each parameter were calculated using GraphPad Prism 4.01 software (GraphPad Software, Inc., San Diego, CA). An analysis of variance with Bonferroni posttest was carried out to examine the differences among strains for each parameter using the same software. Values of *P* < .05 were considered significant. 

## 3. Results

### 3.1. Rectal Temperature, Respiration, and Heart Rates

The mean (±SEM) body weight, morning and evening rectal temperature, respiration, and heart rates recorded for Nagamese, Arunachalee, Mizoram, and Manipur strains are presented in Table 1. The mean body weight differed significantly (*P* < .01) among strains of Mithun (Table 1). Morning rectal temperature of Manipur strain was the highest and differed significantly (*P* < .01) from all other three strains. No significant differences (*P* > .05) were observed for evening rectal temperature among the strains. Manipur strain exhibited significantly (*P* < .01) higher respiration rate (36.6 ± 1.7/min) than other strains. The heart rate of Manipur strain (99.3 ± 12.9/min) was recorded to be the highest, and it differed significantly (*P* < .01) with other three strains whereas the heart rate of Mizoram strain (87.6 ± 6.7/min) differed significantly with Nagamese and Arunachalee strains.

### 3.2. Electrocardiogram

**Figure 1:**
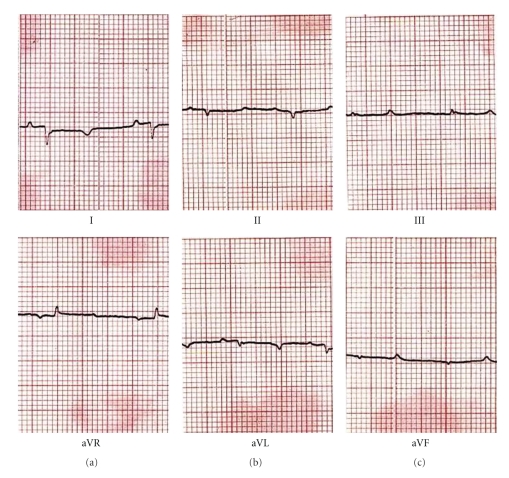
Electrocardiogram of Nagamese mithun (*Bos frontalis*).

**Figure 2:**
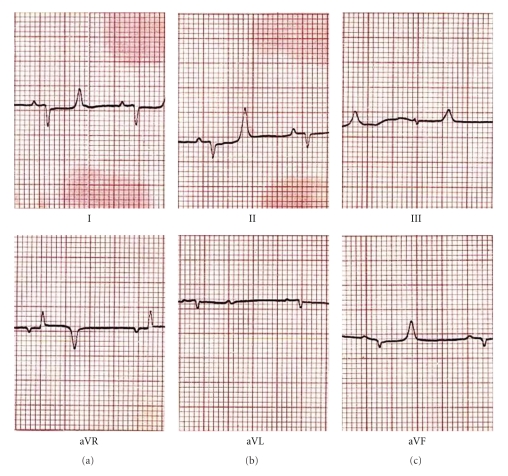
Electrocardiogram of Arunachalee mithun (*Bos frontalis*).

**Figure 3:**
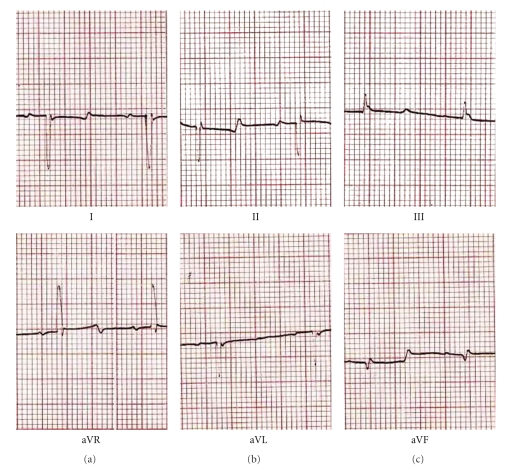
Electrocardiogram of Mizoram mithun (*Bos frontalis*).

**Figure 4:**
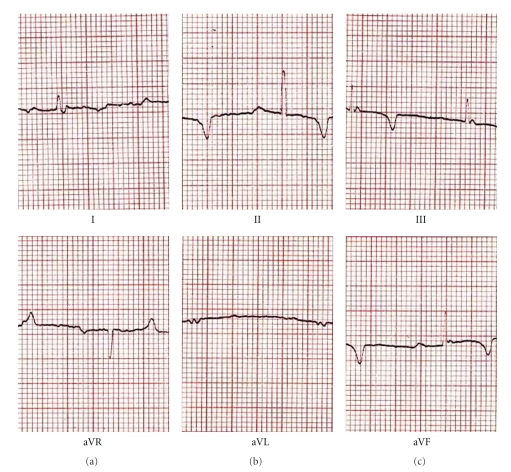
Electrocardiogram of Manipur mithun (*Bos frontalis*).

Electrocardiograms (ECG) of different strains of mithun are presented in Figures 1–4. The amplitude and duration of P wave, QRS complex, T wave of lead–I, and mean electrical axis with axis deviation in frontal plane as derived from ECG tracing are presented in Table 1. Both the amplitude and duration of P wave were found to differ significantly (*P* < .05) among the strains. The highest amplitudes and duration of P wave were observed in Mizoram strain (0.129 ± 0.014 mV and 0.048 ± 0.001 s, resp.) followed by Nagamese (0.121 ± 0.014 mV and 0.055 ± 0.001 s, resp.), Arunachalee (0.120 ± 0.015 mV and 0.048 ± 0.001 s, resp.) and Manipur (0.100 ± 0.001 mV and 0.039 ± 0.002 s, resp.) strains. The highest amplitude (0.494 ± 0.075 mV) and duration (0.055 ± 0.001 s) of QRS complex were recorded in Mizoram strain and it was significantly different (*P* < .05) from other three strains. Higher (*P* < .05) amplitude and duration of T wave were recorded in Arunachalee (0.250 ± 0.025 mV and 0.082 ± 0.003 s, resp.) and in Mizoram (0.300 ± 0.033 mV and 0.085 ± 0.002 s, resp.) strains. 

**Figure 5:**
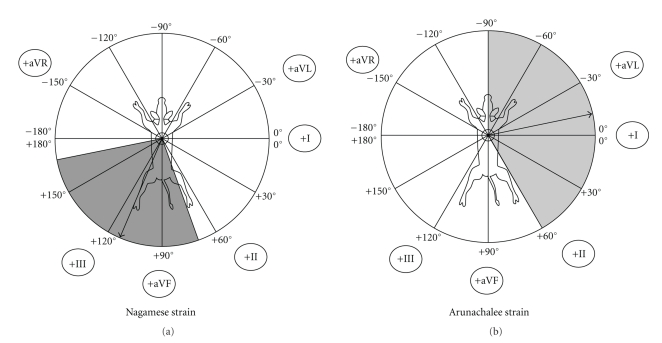
Cardiac electrical axis of Nagamese and Arunachalee Strain of Mithun (*Bos frontalis*).

**Figure 6:**
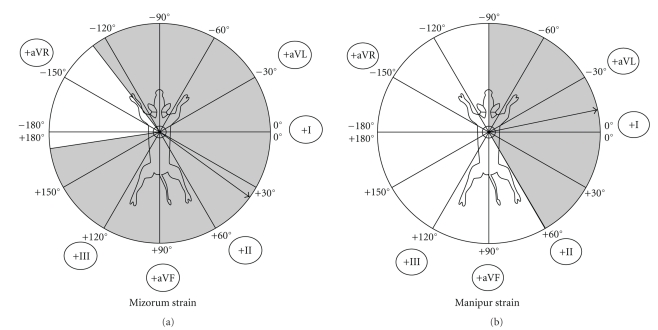
Cardiac electrical axis of Mizoram and Manipur strain of Mithun (*Bos frontalis*).

The electrical axis deviations with mean electrical axis of QRS complex in frontal plane are presented in Figures 5 and 6. The cardiac electrical axis for Nagamese, Arunachalee, Mizoram, and Manipur strains were calculated to be +70° to +167° (+114°), −90° to +60° (−12°), −128° to +170° (+34°) and −90° to +60° (−12°), respectively.

## 4. Discussion

To the best of our knowledge, this is the first report ever that describes the electrocardiogram in clinically healthy mithuns, particularly strain-specific differences in electrocardiograms among different strains. Electrocardiography, a noninvasive technique, is generally a method of choice for evaluating electrical activity of heart and determining irregularities of cardiac rhythm [13, 14]. In small animals and human beings, it can be used for detecting cardiac hypertrophy and dilation [15, 16]. However, in large animals, because of deep penetration of Purkinje fibres in the myocardium, the ECG is not very helpful for detecting cardiac chambers' enlargement [17]. Therefore, in cattle and similarly in mithun, the ECG may mostly be used for detection of cardiac arrhythmias. In order to use a trace for this purpose, it should have clear electrocardiographic waves and complexes. The bipolar limb leads and augmented unipolar limb leads used in the present investigation for mithun were prudent to present electrocardiographic parameters as standard values.

The purpose of the present study was to assess the electrophysiological activity of heart of mithun, and to compare it among the various strains. As social behavior, particularly the temperamental behavior of four different strains of mithun is different since its domestication; hence along with ECG, some extraphysiological parameters, namely, rectal temperature, respiration rate, and heart rate were also studied to assess the cardiovascular physiology of mithun as a whole.

Though the origin of four different strains of mithuns used in the present study was from four respective states of India, they were reared from birth at the farms of the National Research Centre on Mithun in Nagaland State. Hence, the difference in rectal temperature as recorded in different strains of mithun in the present study was probably due to strain variation. The variation in respiration rate among different strains may be due to significant differences in body weight (Table 1). Similarly, higher heart rate in Manipur strain may be due to lower body weight.

The careful and detailed study of different waves of the ECG tracing of mithun in the present investigation showed some unique features which can be compared with other domesticated animals. The variability of the QRS complex in different strains of mithun produces different positions of mean frontal plane of vectors. The narrowest angle (+70° to +167°) of ventricular activation has been found in Nagamese strain with a mean of +114°. This may be due to size of the heart in comparison to their body size. This cardiac axis is similar to feline species [16] considering the positivity of angle of activation. The widest ventricular activation field (−128° to +170°) with a mean electrical axis of +34° as found in Mizoram strain is essentially similar to caprine species [17–19]. This widest ventricular activation process in these caprine and mithun may be due to peculiarity of distribution of conducting fibers. In most mammals, the mean electrical activity or vector during inscription of QRS complex is directed towards the apex of the heart [20]. Moreover, due to high penetration of Purkinje fiber up to epicardium with a higher conduction rate, the ventricular wall excited rapidly with a burst associated with almost simultaneous excitation of all areas of the left ventricle except a small apical epicardial area [21]. The finding of this study indicates a considerable variability of the QRS complex in normal ECG [21]. The identical pattern of electrical axis of Arunachalee and Manipur strains was between −90° to +60° with a mean of −12°, and it was moderately wide and almost similar to that reported for bovine [22]. Although, there are great variations in QRS complex in ruminants including mithuns, some possible explanation for these differences may be due to variation in depolarization pathways, differences in position of the heart within the thorax, or differences within the shape or conductivity of the torso.

In conclusion, electrocardiogram of mithun revealed that the amplitude and duration of P wave, QRS complex, and T wave were different among four different genetic strains of mithun and the electrical axis of QRS complex for Nagamese and Mizoram mithuns are dissimilar to bovine species. 

## Figures and Tables

**Table 1:** Physiological and electrocardiographic parameters (mean ± SEM) of different strains of mithun.

	Nagamese	Arunachalee	Mizoram	Manipur
Physiological Parameter				
Body weight ^†^(kg)	309.3^a^	284.5^ab^	247.1^b^	180.2^c^
	± 88.9	± 72.5	± 48.3	± 54.9
Morning Rectal	38.1^a^	36.7^a^	37.6^a^	39.1^b^
Temperature ^*ζ*^(°C)	± 0.4	± 0.2	± 0.6	± 0.3
Evening Rectal	38.8	38.7	38.9	38.8
Temperature (°C)	± 0.05	± 0.5	± 0.06	± 0.03
Respiration rate ^§^	29.0^a^	32.5^a^	30.0^a^	36.6^b^
(per minute)	± 0.5	± 1.7	± 0.9	± 1.7
Heart rate ^#^	83.2^ab^	78.0^ac^	87.6^b^	99.30^d^
(per minute)	± 6.9	± 4.09	± 6.79	± 12.9
Electrocardiographic parameter (lead – I)				
Amplitude of P ^¥^	0.12^a^	0.12^a^	0.13^b^	0.10^c^
(mV)	± 0.01	± 0.02	± 0.01	± 0.00
Duration of P^*€*^	0.06^a^	0.05^a^	0.06^b^	0.04^c^
(s)	± 0.001	± 0.001	± 0.002	± 0.002
Amplitude of	0.37^a^	0.36^a^	0.50^b^	0.28^c^
QRS ^∞^(mV)	± 0.07	± 0.03	± 0.0 8	± 0.08
Duration of	0.045^a^	0.044^a^	0.055^b^	0.042^a^
QRS ^*∂*^(s)	± 0.001	± 0.002	± 0.001	± 0.001
Amplitude of T*^¶^*	0.21^a^	0.25^b^	0.30^c^	0.20^a^
(mV)	± 0.024	± 0.025	± 0.033	± 0.024
Duration of T^*θ*^	0.06^a^	0.08^b^	0.09^b^	0.06^a^
(s)	± 0.001	± 0.003	± 0.002	± 0.001
Mean Electrical	+114°	−12°	+34°	−12°
axis in° (frontal plane)	(+70° to + 167°)	(−90° to +60°)	(−128° to + 170°)	(−90° to +60°)

*P* value is as follows, respectively

^†^ *P* < .01, ^*ζ*^ *P* < .01, ^§^ *P* < .05, ^#^ *P* < .05, ^*¥*^ *P* < .05, ^*€*^ *P* < .01, ^**∞**^ *P* < .05, ^∂^ *P* < .05, ^*¶*^ *P* < .05, ^*θ*^ *P* < .05

^a, b, c, d^Mean bearing different superscript in the same row differ significantly.
